# Patient satisfaction with anaesthesia services and associated factors at the University of Gondar Hospital, 2013: a cross-sectional study

**DOI:** 10.1186/s13104-015-1332-4

**Published:** 2015-08-26

**Authors:** Endale Gebreegziabher Gebremedhn, Wubie Birlie Chekol, Wubet Dessie Amberbir, Tesera Dereje Flatie

**Affiliations:** Department of Anaesthesia, School of Medicine, Gondar College of Medicine and Health Sciences, University of Gondar, P.O. Box 196, Gondar, Ethiopia

**Keywords:** Patient satisfaction, Anaesthesia service, Associated factors

## Abstract

**Background:**

Patient satisfaction is the degree of fulfilling patients’ anticipation which is an important component and quality indicator in anaesthesia service. It can be affected by anaesthetist patient interaction, perioperative anaesthetic management and postoperative follow up. No previous study conducted in our setup. The aim was to assess patient satisfaction with anaesthesia services and associated factors.

**Methods:**

Institutional based cross sectional study was conducted from April 15–30, 2013 at the University of Gondar referral and teaching hospital. All patients who were operated upon both under general and regional anaesthesia during the study period were included. Standardized questionnaire used for postoperative patient interview. Data was entered and analyzed using Statistical Package for Social Sciences (SPSS) window version 20. Chi Square test used to assess the association between each factor and the overall satisfaction of patients. The proportion of patients who said they were satisfied with anaesthesia services was presented in percentage.

**Results:**

A total of 200 patients were operated upon under anaesthesia during the study period. Of these, a total of 156 patients were included in this study with a response rate of 78 %. The overall proportion of patients who said they were satisfied with anaesthesia services was 90.4 %. Factors that affected patient satisfaction negatively (dissatisfaction level and *p* value) were general anaesthesia (12.6 %, *P* = 0.046), intraoperative awareness (50 %, *P* = <0.001), pain during operation (61.1 %, *P* = <0.001), and pain immediately after operation (25 %, *P* = <0.001) respectively.

**Conclusion and recommendation:**

Patient satisfaction with anaesthesia services was low in our setup compared with many previous studies. Factors that affected patient satisfaction negatively may be preventable or better treated. Awareness creation about the current problem and training need to be given for anaesthetists.

## Background

Measuring the degree of patient satisfaction can be achieved with a variety of tools such as post operative visits and questionnaires [1, 2]. Many factors contribute to patient satisfaction including accessibility and convenience of services that depend upon institutional structures, interpersonal relationships, competence of health professionals and patient expectations and preferences [3].

Poor quality of anaesthesia services may discourage patient from using available services. Because health concerns are among the most important of human concerns [4, 5]. Patient satisfaction is the balance between expectations and perceptions of what was received. If there was concern, staff must continue to identify, monitor and modify factors that may improve it [6]. Patient satisfaction with anaesthesia services remains the best way to assess the outcome from the patients’ point of view [7]. But it is difficult to measure satisfaction especially during the preoperative and intra operative periods [8–12].

There is inconsistency regarding patient satisfaction report which may be attributed to differences in institutional structures, interpersonal relationships, competence of health professionals, patient expectations and preferences, and variations in tools that used for data collection.

Of patients who operated upon under general anesthesia and local anesthesia, 87 % of patient was satisfied, 0.5 % dissatisfied and 12.5 % had no opinion [13]. Whereas another study revealed that 54 % of patients achieved an overall satisfaction less than 85 %, and female, educated and American Society of Anesthesiologists physical status (ASA) 1–2 patients were less satisfied [14].

From a study in Australia, patient satisfaction and other predetermined outcomes such as nausea, vomiting, pain and complication were assessed. The overall level of satisfaction was high (96.8 %), 2.3 % patients were somewhat dissatisfied and 0.9 % were dissatisfied with their anaesthetic care [15].

In a study done in Japan patients, 3.9 % had dissatisfaction with anaesthesia. The rates of dissatisfaction were higher in women than men and in spinal anaesthesia than in general anesthesia, and were observed mostly in patients aged from 20 to 39 years [16]. In the study done in British perception of anaesthetist, patient satisfaction with continuity of personal care by the anaesthetist were significantly increased by the introduction of a single post operative visit by the anaesthetist compared with no visit at all [17].

A study conducted in Taiwan showed a higher score of satisfaction in anaesthesia inclusive of waiting time for surgery in the operation room, attitude towards an anaesthetic staffs and during post operative visit in the management of complications of patients who were offered small video teaching in comparison with patients of traditional preoperative visit [18]. The clothing worn by the anaesthetist did not affect patients’ satisfaction [19]. Studies showed that 62 % recalled being very anxious and afraid of anaesthetic as well as the operation and 40 % said that their recall before operation was of a mask covering their face [20].

Patient involvement in decision making increases satisfaction with anaesthesia services [21]. A study conducted in Kashihara showed that the most undesirable postoperative outcomes were vomiting, nausea, sore throat, postoperative pain and memory of extubation [22]. A study in Greek showed that the overall patient satisfaction in anaesthesia services rates in the range of 96.3–98.6 % [23].

Patient satisfaction is one of the indicators of the quality of health care provision. The department of anaesthesia at the University of Gondar referral and teaching hospital provides health services for millions of patients. As far as our search and knowledge is concerned, there was no previous research done in the study area that may reveal the level of patient satisfaction. Therefore, the aim of this study was to determine the level of patient satisfaction with anaesthesia services and associated factors.

## Methods

*Study design* Quantitative cross sectional study design was used.

*Study area and period* The University of Gondar referral and teaching hospital is found in North Gondar Administration Zone, Amhara Regional state which is far from about 727 km Northwest of Addis Ababa (the capital city of Ethiopia). It is one of the largest hospitals in the country which provides health services for more than five million people in the catchment area. The hospital has 500 hundred beds, seven operation theatres and one medical intensive care unit. According to the annual report of anaesthesia department, 5695 patients were operated upon under anaesthesia in 2013. The study was conducted from April 15–30, 2013.

*Study population* All emergency and elective patients (minor, major, surgery, gynecology, obstetric and ophthalmology) who were operated upon under anaesthesia during the study period were included. Patients less than 18 years old, patients who were unconscious 24 h after operation, patients who were discharged before 24 h and patient who refused to participate in the study were excluded.

### Variables of the study

*Dependent variables* Proportion of patients who would say they were satisfied.

*Related variables* Socio-demographic variables: sex, age, educational Status.

*Factors related with surgery, anaesthesia and disease status* Type of surgery, type of anaesthesia and ASA status.

*Factors related with preoperative anaesthesia evaluation* Self introduction of anaesthetist, way of approach, give chance for the patient to choose type of anesthesia, give chance for the patient to ask question, giving adequate information about the type of anesthesia.

*Factors related with reception in the operation theatre and intra operative management* Reception, pain on induction, privacy, awareness, pain during operation and immediately after operation.

*Factors related with postoperative anaesthetic revisit* Anaesthetist revisit and number of visits.

*Factors related with anaesthetic related discomforts and complications* Sore throat, discomfort, depression, pain, nausea, vomiting and shivering.

### Operational definitions

#### Patient satisfaction

The proportion of patients who would say they were satisfied with anaesthesia services at the University of Gondar hospital would be classified as satisfactory if the proportion of patients who would say they were satisfied would be >95 % (average from previous studies).

#### Major operation

A major operation was defined as any invasive operative procedure in which a more extensive resection is performed, e.g., a body cavity is entered, organs are removed, or normal anatomy is altered in general, if a mesenchymal barrier was opened (pleural cavity, peritoneum, meninges).

#### Minor operation

A minor operation was defined as any invasive operative procedure in which only skin or mucus membranes and connective tissue are resected, e.g., vascular cutdown for catheter placement or implanting pumps in subcutaneous tissue.

#### Intraoperative awareness

A recalled event occurring during anaesthesia/surgery that was confirmed (or otherwise) by the attending personnel present in the operating room was considered as awareness [24].

#### Postoperative depression

Defined as having unexplained feelings of sadness and hopelessness after a surgery.

#### Postoperative nausea and vomiting

When the patient experiences at least one or more episode of nausea or vomiting and/or both within 24 h of operation.

#### Postoperative sore throat

When the patient experiences pain during swallowing (liquid or solid food) or develops hoarseness of voice within 24 h after operation among those patients who managed with general anaesthesia with tracheal intubation.

*Sample size and sampling procedure* All elective and emergency patients (minor, major, surgery, gynecology, obstetric, ophthalmology) who were operated upon under anaesthesia during the study period were included.

*Data collection procedure* A pre-tested Amharic version questionnaire and check list used for data collection. The Amharic version questionnaire was first developed and translated into English language by English language experts. The English version questionnaire translated back into Amharic language by Amharic language experts. The pre-test was done on twenty patients in other hospital and corrections were made before the data collection. The data collected on socio-demographic characteristics and factors associated with preoperative, intraoperative, postoperative anaesthesia management of patients after 24 h of operation using face to face interview. Two BSc holder anaesthetists were involved in data collection after training.

*Data analysis and interpretation* SPSS window version 20 was used for data entry and analysis. Chi square test used to assess the association between each factor and the overall satisfaction of patients. The findings are presented in percentages and tables used for presentation of descriptive statistics.

*Ethical consideration* Ethical approval was obtained from the School of Medicine (Gondar College of Medicine and Health Sciences) ethical review committee since our department is under school of medicine. There is no other independent ethical approval body other than from the respective schools, institutes and Vice President for Research and Community Services ethical approval committees where all of them are parts of the University of Gondar. Oral informed consent was obtained from the study subjects and the aim of the research was explained to the patients and those who were not voluntary were excluded. Confidentiality was ensured by avoiding personal identifications, keeping questionnaires and checklists locked. Those patients with pain during induction of anaesthesia as well as the intraoperative and postoperative periods, postoperative nausea and vomiting and intraoperative awareness were reassured and their responsible anaesthetists, nurses and physicians were informed about the problem.

## Results

### Sociodemographic characteristics of the study participants

A total of 200 patients were operated upon under anaesthesia during the study period. Of these, 4 (2 %) refused, 20 (10 %) were <18 years old, 10 (5 %) were discharged in the first 24 h after surgery, and 10 (5 %) were unconscious postoperatively.

A total of 156 patients were included in this study with a response rate of 78 %. The majority of study subjects, 81 (51.9 %) were females and the rest 75 (48.1 %) were males. Most of the respondents 83 (53.2 %) were in the age group of 18–29 years, 47 (30.1 %) were in the age group of 30–49 years, 17 (10.9 %) were in the age group of 50–65 years and 9 (5.8 %) were in the age group of >65 years old respectively. The male to female ratio was (0.9:1) and the mean age of the study subjects was 33 years (SD = 14.8), with minimum and maximum value 18 and 81 years respectively. Of the participants, 62 (39.7) were unable to read and write, 45 (28.8 %) were able to read and write, 35 (22.4 %) were 9–12 grades and 14 (9 %) were above grade 12.

### Type of anaesthesia, surgery and disease status

Of a total of 156 patients, 111 (71.2 %) were operated upon under general anesthesia and 45 (28.8 %) were operated under regional anesthesia. Hundred thirty-one (84 %) were major surgeries and 25 (16 %) were minor surgeries. From these patients 107 (68.6 %) were ASA 1, 48 (30.8) ASA 2 and 1 (0.6 %) ASA3 (Table 1).

**Table 1:** Type of anesthesia, surgery and disease status, University of Gondar teaching hospital, 2013 (N = 156)

Factor	Frequency	Percentage
Type of anaesthesia		
General	111	71.2
Regional	45	28.8
Type of surgery		
Major	131	84
Minor	25	16
ASA status		
ASA 1	107	68.6
ASA 2	48	30.8
ASA 3	1	0.6

### Preoperative anaesthetic evaluation

From 156 patients, the anaesthetist introduced him/herself to 80 (51.3 %) patients, 153 (98.1 %) of participants explained that the approach of the anaesthetists were good, 79 (50.6 %) of the respondents were not given adequate information about anesthesia, 114 (73.1 %) of respondents were not given a chance to choose type of anesthesia and 119 (76.3 %) of respondents were not given a chance to ask questions about anesthesia (Table 2).

**Table 2:** Preoperative anaesthetic evaluation, University of Gondar teaching hospital, 2013 (N = 156)

Factor	Frequency	Percentage
Anaesthetist introduced him or her self		
Yes	80	51.3
No	76	48.7
Anaesthetists approach		
Good	153	98.1
Bad	3	1.9
Adequate anaesthesia information		
Yes	77	49.4
No	79	50.6
Chance to choose type of anaesthesia		
Yes	42	26.9
No	114	73.1
Chance to ask question		
Yes	37	23.7
No	119	76.3

### Reception in the operating theatre and intraoperative patient management

Out of 156 patients, 154 (98.7 %) explained that the reception of the anaesthetist was good, 151 (96.8 %) of respondent’s privacy was kept by the anaesthetists, 14 (94.2 %) of participants did not feel pain during induction of anaesthesia, 89 (57.1 %) of respondents did not recall anything intraoperatively. One hundred and thirty-eight (88.5 %) and 104 (66.7 %) patients had no pain during and immediately after operation respectively (Table 3).

**Table 3:** Reception in the theatre and intraoperative management, University of Gondar teaching hospital, 2013 (N = 156)

Factor	Frequency	Percentage
Reception		
Good	154	98.7
Bad	2	1.3
Privacy		
Yes	151	96.8
No	5	3.2
Pain during induction		
Yes	9	5.8
No	147	94.2
Awareness		
Yes	22	14.1
No	134	85.9
Intraoperative pain		
Yes	18	11.5
No	138	88.5
Pain immediately after operation		
Yes	52	33.3
No	104	66.7

### Postoperative anaesthetist revisit

Out of 156 patients, 125 (80.1 %) were not visited postoperatively, 19 (12.2 %) and 12 (7.7 %) were visited once and greater or equal to twice respectively. All patients who were revisited postoperatively were treated for their complaints (Table 4).

**Table 4:** Postoperative anaesthetist revisit, University of Gondar teaching hospital, 2013 (N = 156)

Factor	Frequency	Percentage
Did the anaesthetist visit you after operation?		
Yes	31	19.9
No	125	80.1
Postop re-visit		
No	125	80.1
Once	19	12.2
≥twice	12	7.7
Did he/she treat your complain		
Yes	31	19.9
No	125	80.1

### Postoperative anaesthetic related discomfort and complication

Of a total of 156 study participants, 34 (21.8 %), 35 (22.4 %), 25 (16 %) and 42 (26.9 %) of patients had postoperative nausea and vomiting, shivering, depression and sore throat respectively (Table 5).

**Table 5:** Postanesthetic related discomfort and complications, University of Gondar teaching hospital, 2013 (N = 156)

Factor	Frequency	Percentage
Nausea and vomiting		
Yes	34	21.8
No	122	78.2
Shivering		
Yes	35	22.4
No	121	77.6
Depression		
Yes	25	16
No	131	84
Sore throat		
Yes	42	26.9
No	114	73.1

### Patient satisfaction with anaesthesia services

The overall proportion of patients who said they were satisfied with anaesthesia services in this study was 90.4 %. Out of 75 male patients, 66 (88 %) were satisfied and 9 (12 %) were dissatisfied. Whereas from 81 female patients, 75 (92.6 %) were satisfied and 6 (7.4 %) dissatisfied (male vs. female, *P* = 0.0.331). From 62 illiterate patients, 54 (87.1 %) were satisfied and 8 (12.9 %) dissatisfied. Of 45 patients who can read and write, 40 (88.9 %) were satisfied and 5 (11.1 %) dissatisfied. Out of 35 patient with grades 9–12, 34 (97.1 %) were satisfied and 1 (2.9 %) dissatisfied. Whereas among 12 patients with qualification of >grade 12, 13 (92.8 %) were satisfied and 1 (7.1 %) dissatisfied (illiterate vs. non illiterate, *P* = 0.419).

Of 111 patients who managed under general anaesthesia, 97 (87.4 %) were satisfied and 14 (12.6 %) dissatisfied. Whereas of 45 regional cases, 44 (97.8 %) were satisfied and 1 (2.2 %) dissatisfied (GA vs. RA, *P* = 0.046). From 131 patients who had major operation, 119 (90.8 %) were satisfied and 12 (9.2 %) dissatisfied. On the other hand, of 25 minor operation cases, 22 (88 %) were satisfied and 3 (12 %) were dissatisfied (major vs. minor, *P* = 0.659).

Out of 77 (49.7 %) patients who had adequate information about anaesthesia, 70 (90.9 %) were satisfied and 7 (9.1 %) were dissatisfied. Whereas among 79 patients who did not get information, 71 (89.8 %) were satisfied and 8 (10.1 %) were dissatisfied (adequate information vs. inadequate information, *P* = 0.826).

Twenty-two out of 111 patients who operated upon under general anaesthesia experienced intraoperative awareness where 11 (50 %) of the patients dissatisfied (satisfied vs. dissatisfied, *P* = <0.001). Eighteen patients experienced pain during operation which dissatisfied 11 (61.1 %) patients (satisfied vs. dissatisfied, *P* = <0.001). In addition, 52 patients experienced pain immediately after operation. Of these, 13 (25 %) patients dissatisfied (satisfied vs. dissatisfied, P = <0.001) (Table 6).

**Table 6:** The effect of factors on the overall satisfaction of patients and Chi-Square test, 2013

Factor	Total number of patients (n)	Patients satisfied n (%)	Patients dissatisfied n (%)	P value
Anaesthetist self introduction				
Yes	80	71 (88.7 %)	9 (11.3 %)	0.477
No	76	70 (92.1 %)	6 (7.9 %)	
Anaesthetist’s approach to you				
Good	153	138 (90.2 %)	15 (9.8 %)	0.568
Bad	3	3 (100 %)	0 (0 %)	
Chance to choose type of anaesthesia				
Yes	42	38 (90.5 %)	4 (9.5 %)	0.981
No	114	103 (90.4 %)	11 (9.6 %)	
Chance for asking questions				
Yes	37	36 (97.3 %)	1 (2.7 %)	0.102
No	119	105 (88.2 %)	14 (11.8 %)	
Reception in the theatre				
Good	154	140 (90.9 %)	14 (9.1 %)	0.051
Bad	2	1 (50 %)	1 (50 %)	
Patient privacy in theatre				
Good	151	136 (90.1 %)	15 (9.9 %)	0.459
Bad	5	5 (100 %)	0 (0 %)	
Did you feel pain during induction of anaesthesia?				
Yes	9	9 (100 %)	0 (0 %)	0.313
No	147	132 (89.8 %)	15 (10.2 %)	
Did you remember anything during operation (general anaesthesia)?				
Yes	22	11 (50 %)	11 (50 %)	<0.001
No	89	85 (95.5 %)	4 (4.5 %)	
Did you feel pain during operation?				
Yes	18	7 (38.9 %)	11 (61.1 %)	<0.001
No	138	134 (97.1 %)	4 (2.9 %)	
Did you experience pain immediately after operation?				
Yes	52	39 (75 %)	13 (25 %)	<0.001
No	104	102 (98.1 %)	2 (1.9 %)	
Postoperative anaesthetist visit				
Yes	31	27 (87.1 %)	4 (12.9 %)	0.488
No	125	111 (88.8 %)	14 (11.2 %)	
No of postoperative anaesthetist visit				
0	125	111 (88.8 %)	14 (11.2 %)	0.121
1	19	15 (78.9 %)	4 (21.1 %)	
≥2	12	12 (100 %)	0 (0 %)	
Did the anaesthetist treat your complain during visit?				
Yes	31	27 (87.1 %)	4 (12.9 %)	0.488
No	125	111 (88.8 %)	14 (11.2 %)	
Did you have postoperative sore throat?				
Yes	42	36 (85.7 %)	6 (14.3 %)	0.230
No	114	105 (92.1 %)	9 (7.9 %)	
Did you have postoperative depression?				
Yes	25	25 (100 %)	0 (0 %)	0.075
No	131	116 (88.5 %)	15 (11.5 %)	
Did you experience PONV?				
Yes	34	33 (97.1 %)	1 (2.9 %)	0.135
No	122	108 (88.5 %)	14 (14.5 %)	
Did you have postoperative shivering?				
Yes	35	34 (97.1 %)	1 (2.9 %)	0.124
No	121	107 (88.4 %)	14 (11.6 %)	

## Discussion

This study showed that the overall proportion of patients who said they were satisfied with anaesthesia services at the University of Gondar teaching hospital was 90.4 %. This finding was low compared with other studies [2, 4, 15, 20, 22]. This could be due to poor preoperative evaluation and preparation, intraoperative and postoperative patient management in our setup.

In this study, the level of satisfaction of males was 88 % and that of females was 93.6 %. But a study done in India showed that females were less satisfied [1]. This difference could be due to only short-stay surgical inpatients were included in Indian study.

In our study, those who were unable to read and write had low level of satisfaction (87 %), but a study conducted in Saudi Arabia showed that the educated were less satisfied [14]. This discrepancy might be due to a difference in study design. We interviewed patients after operation which could enable to explore the problem properly but self administered questionnaire was used in Saudi Arabia.

As age increases patient satisfaction rate decreased, but in the study conducted in Japan patient dissatisfaction rate was high at the age of 20-39 years old. This difference might be due to small number of aged patients were included in our study. On the contrary, in Japan they included large number of aged patients [16].

In this study, more patients operated upon under regional anesthesia were satisfied than those operated upon under general anesthesia (general vs. regional, P = <0.001), but in the study conducted in Japan patients who operated with regional anaesthesia were dissatisfied [16]. This discrepancy might be due to small number of patients were given regional anesthesia and of these, the majority were managed under spinal anaesthesia in our setup. On the other hand, most patients were operated upon under peripheral block which might cause pain during the block that may lead to dissatisfaction in the case of Japan.

Patients who had post operative visit (100 %) were more satisfied than those who did not get visit at all (visit vs. no visit, *P* = 0.488). This finding was similar with a study conducted in Austria [17]. This could be because of reassurance of patients and patients might get treatment for their complaint at the right time.

Perioperative pain relief is the one of the key reflection of the level of patient satisfaction with anaesthesia service provided in hospitals. Eighteen (11.5 %) patients had pain during operation. From these, 11 (61.1 %) patients were dissatisfied (pain vs. no pain, *P* = <0.001). On the other hand, 52 out of 156 (33.3 %) patients had pain immediately after operation. Of these, 13 (25 %) were dissatisfied (pain vs. no pain, *P* = <0.001). This could be due to the substandard perioperative pain management practice in our hospital which is consistent with another study [20].

Only 42 (26.9 %) patients had a chance to choose anaesthesia, from these 4 (9.5 %) were dissatisfied (chance vs. no chance, *P* = 0.981). On the other hand, 114 (73.1 %) patients did not get a chance to choose anaesthesia, from these 45 (36.5 %) were dissatisfied. Out of 156 participants, 37 (23.7 %) got a chance for questioning, of these 1 (2.7 %) were dissatisfied. Whereas 119 (76.3 %) did not get a chance for questioning and from these, 14 (11.8 %) patients were dissatisfied (chance vs. no chance, *P* = 0.102) which might be due to anxiety. These findings showed that patient involvement in decision making seems to have a positive impact on patient satisfaction with anaesthesia service which is in accordance with another study [21].

Among 42 (26.9 %) patients who had postoperative sore throat, 6 (14.2 %) patients were dissatisfied. On the other hand, of 114 (73.1 %) patients that had no sore throat, 9 (7.8 %) patients were dissatisfied with anesthesia services (sore throat vs. no sore throat, *P* = 0.230). These findings were similar with other study [22].

Twenty-two out of 111 patients who operated upon under general anaesthesia experienced intraoperative awareness where 11 (50 %) of the patients were dissatisfied (awareness vs. no awareness, *P* = <0.001). Our finding was high compared with most previous studies [24–27]. This may be attributed to lack of optimal perioperative patient management including pain management experience of professionals in our set up compared with the developed countries.

### Limitation of the study

The limitation of the study was that the sample size was small due to time constraint, the short time elapsed between anaesthesia and assessment of satisfaction, and the use of a questionnaire with dichotomous answers.

## Conclusion

The overall proportion of patients who said they were satisfied with anaesthesia services was low at the University of Gondar referral and teaching hospital.

General anaesthesia, pain during operation, intraoperative awareness, and pain immediately after operation were the determinant factors for patient dissatisfaction with anaesthesia services in our set up. These factors may be preventable or better treated.

### Recommendation

The problem should be given emphasis and awareness creation need to be made for all anaesthetists. Anaesthetists need to give a chance for the patient to ask a question and choose type of anaesthesia and should tell any possible side effects and potential complications related to anaesthesia. Further study should be conducted with large sample size.

## Appendix: English version questionnaire

See Tables 7, 8, 9, 10, 11.

**Table 7:** Socio-demographic variables

S. no.	Questions	Possible response
101.	Sex	A. Male
		B. Female
102.	Age	___ years old
103.	Educational status	A. Unable to read and write
		B. Able to read and write
		C. Grade1-8
		D. Grade 9–12
		E. >Grade 12
104.	Type of anesthesia	A. General
		B. Regional
105.	Type of surgery	A. Minor
		B. Major
106.	American Society of Anesthesiologists (ASA) status	A. 1
		B. 2
		C. 3
		D. 4

**Table 8:** Factors related with preoperative anaesthetic evaluation

S. no.	Questions	Possible response
201.	Did the anesthetist introduce him/herself to you?	1. Yes
		2. No
202.	How did you get the anaesthetist’s approach to you?	1. Good
		2. Bad
203.	Did the anaesthetist give to you adequate information about anaesthesia?	1. Yes
		2. No
204	Did you get a chance to choose the type of anesthesia?	1. Yes
		2. No
205.	Did the anesthetist give to you a chance to ask questions?	1. Yes
		2. No

**Table 9:** Factors related with perception in the operating theatre and intra operative management

S. no.	Questions	Possible response
301.	How was the reception of the anaesthetist in the operation theatre?	1. Good
		2. Bad
302.	Did you feel pain during induction of anaesthesia?	1. Yes
		2. No
303.	Did the anaesthetist consider your privacy in the operation theatre?	1. Yes
		2. No
304.	Did you feel pain during operation?	1. Yes
		2. No
305.	Did you feel pain immediately after operation?	1. Yes
		2. No
306.	Did you remember anything during the intraoperative period (for general anaesthesia)?	1. Yes
		2. No
307.	If your answer is ‘’Yes’’ for Q306	
	1. What is the last thing you remember before going to sleep?	1.______________
	2. What is the first thing you remember after waking up? (*we used this question for analysis*)	2.______________
	3. Do you remember anything between going to sleep and waking up? (possible answers: yes, no); If you do, what is it? (light, sound, pain, dyspnoea, etc.)	3.______________
	4. Did you dream during surgery? (yes, no), If so, what did you dream about?	4.______________
	5. What was the worst thing that you remember about surgery and anaesthesia?	5.______________

**Table 10:** Factors related with postoperative anaesthetist revisit

S. no.	Questions	Possible response
401.	Did the anaesthetist visit you after operation?	1. Yes
		2. No
402.	If your answer is “yes” for Q 40, how many times?	1. Once
		2. Twice
		3. Three times and more
403.	Did the anaesthetits treat your complain during the visit?	1. Yes
		2. No

**Table 11:** Factors related with postoperative anaesthetic related discomfort and complications

No.	Questions	Possible response
501.	Did you have pain during swallowing of food or hoarseness of voice after operation?	1. Yes
		2. No
502.	Were you depressed postoperatively?	1. Yes
		2. No
503.	Did you experience any episode of nausea and/or vomiting after operation?	1. Yes
		2. No
504.	Was there any shivering?	1. Yes
		2. No
505.	How was your overall satisfaction with anaesthesia services?	1. Good
		2. Bad

## Abbreviations

ASA: American Society of Anesthesiologists
SPSS: statistical package for social sciences
PONV: postoperative nausea and vomiting
